# Supplementary material for: SARS-CoV-2 Spike Protein and Neutralizing Anti-Spike Protein Antibodies Modulate Blood Platelet Function
Source: Int J Mol Sci. 2023 Mar 10;24(6):5312. doi: 10.3390/ijms24065312 (PMC10049216; doi:10.3390/ijms24065312)
Supplement: Supplementary file 1 [file ijms-24-05312-s001.zip › ijms-2210005-supplementary.pdf]

SARS-CoV-2 Spike Protein and Neutralizing Anti-Spike Protein Antibodies Modulate Blood Platelet Function

Boguslawa Luzak, Marcin Rozalski, Tomasz Przygodzki, Magdalena Boncler, Dagmara Wójkowska, Marcin Kosmalski, Cezary Watala

Table S1. CD62 and PAC1 binding in platelets after induction with agonists at high concentrations in High and Low Ab-group.

	**High Ab-group (n=13)**	**Low Ab-group (n=18)**
**PAC1 binding** [fluorescence median]		
no agonist		
Collagen 10 µg/ml	765 (692; 859)	725 (668; 799)
ADP 10 µM	1201 (1054; 1454)	1279 (1034; 1629)
TRAP 8 µM	2373 (1942; 2715)*	1951 (1367; 2242)
	1799 (1396; 2404)	1236 (1049; 2056)
**CD62 exposure** [fluorescence median]		
no agonist	853 (691; 949)	846 (694; 991)
Collagen 10 µg/ml	2107 (1963; 2655)	2518 (1861; 3310)
ADP 10 µM	1946 (1550; 2378)	1671 (1361; 2097)
TRAP 8 µM	3207 (2888; 4018)	2773 (2382; 4389)

Data presented as median and quartiles (Q1; Q3). The significance of differences between High Ab-group vs. Low Ab-group was estimated using the bootstrap-boosted Mann–Whitney U-test, *P<0.05.

Table S2. Effect of spike protein on the fibrinogen receptor (GPIIbIIIa) activation (PAC1 binding) and CD62 exposure on washed platelets.

	PAC1-positive fraction [%]		CD62-positive fraction [%]	
	**control**	**spike**	**control**	**spike**
no agonist	2.4 (0.9; 3.9)	2.3 (1.5; 5.8)	7.9 (1.9;18.7)	16.7 (6.0; 19.7)
Collagen [10 µg/ml]	12.8 (7.6; 19.7)	13.2 (7.3; 27.8)	33.1 (24.4; 55.6)	36.2 (23.1; 54.1)
Collagen [20 µg/ml]	18.2 (12.8; 35.5)	16.7 (11.5; 45.2)	40.7 (34.9; 82.1)	44.5 (25.7; 80.2)
ADP [5 µM]	25.7 (14.9; 30.1)	26.6 (7.8; 32.9)	34.6 (33.0; 43.0)	41.3 (36.7; 47.1)
ADP [10 µM]	34.7 (29.4; 46.5)	33.8 (15.5; 52.6)	45.1 (41.2; 56.1)	45.6 (41.8; 57.0)

Data presented as median and quartiles (Q1; Q3), n=9. There was no significant difference between spike and control samples. Data were analyzed with bootstrap-boosted Wilcoxon signed-rank test.

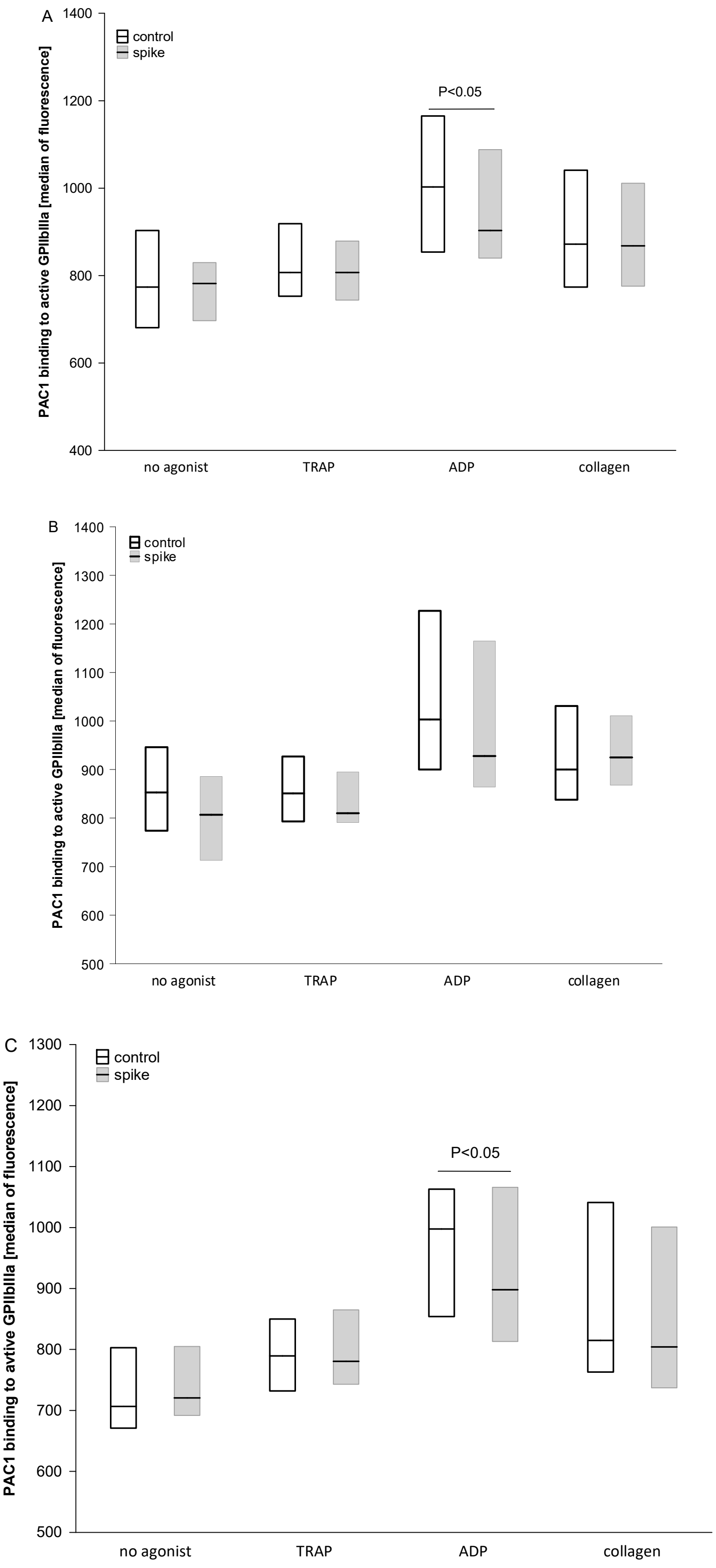

Figure S1. Effect of spike protein on fibrinogen receptor GPIIbIIIa activation (binding of PAC1) in platelets. Data are shown as the median and interquartile range (Q1; Q3). The analysis was performed in all participant groups, n=31 (**A**), in the High Ab-group, n=13 (**B**), and in the Low Ab-group, n=18 (**C**). A significant reduction in the presence of spike protein was observed for ADP-stimulated platelets total participant group and Low Ab-group (P<0.05). The statistical significance of the differences between control vs. spike sample was estimated with the bootstrap-boosted paired Student’s t-test.

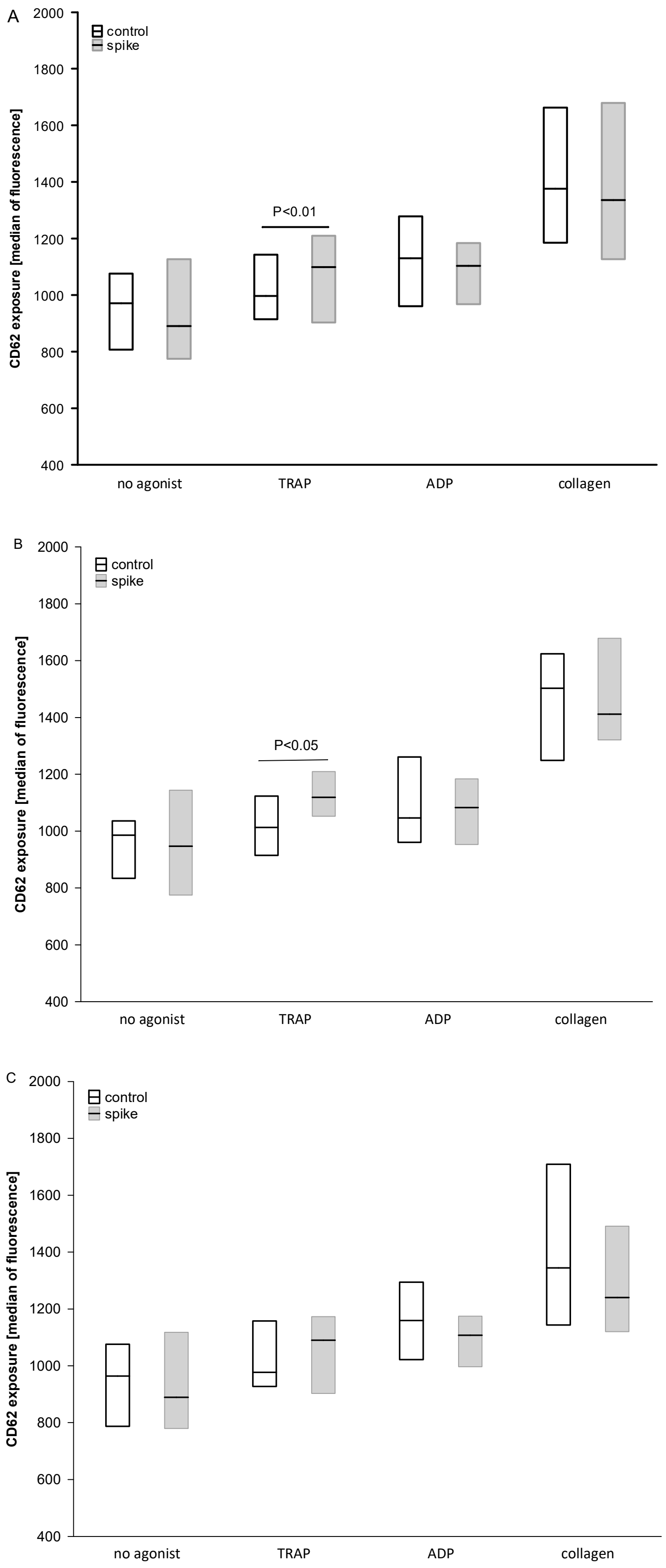

Figure S2. The effect of spike protein on CD62 exposure on the platelet surface. Data are shown as median and interquartile range (Q1; Q3). The analysis was performed in all participants, n=31 (**A**), in the High Ab-group, n=13 (**B**), and in the Low Ab-group, n=18 (**C**). A significant increase in CD62 exposure in spike-treated platelets was observed for TRAP-induced samples in total group or in High Ab-group (P<0.05). The statistical significance of the differences between control vs. spike sample was estimated with the bootstrap-boosted paired Student’s t-test.

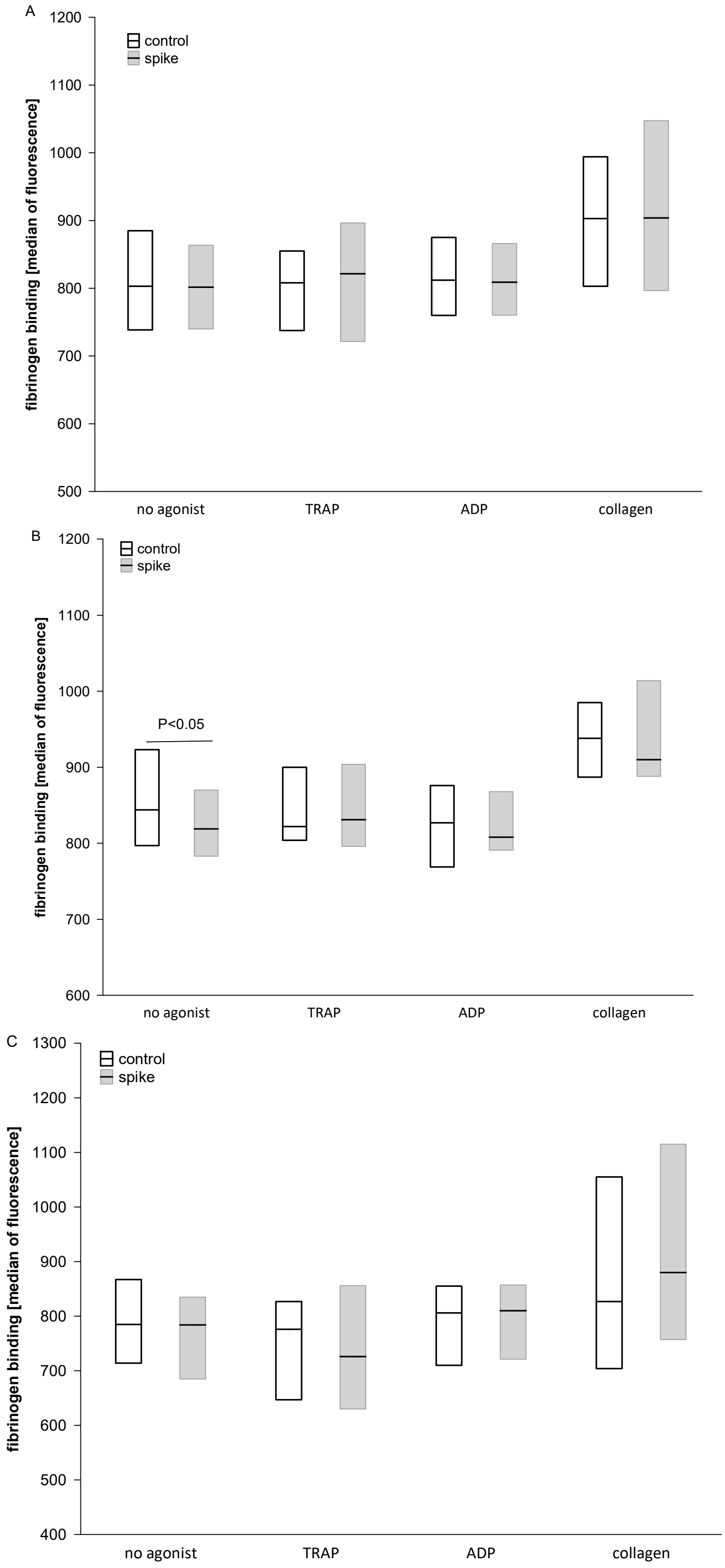

Figure S3. The effect of spike protein on the fibrinogen binding to platelets. Data are shown as median and interquartile range (Q1; Q3). The analysis was performed in all participants, n=31 (**A**), in the High Ab-group, n=13 (**B**), and in the Low Ab-group, n=18 (**C**). A significant increase in fibrinogen binding to spike-treated platelets was observed for non-agonist sample in High Ab-group (P<0.05). The statistical significance of the differences between the control and spike samples was estimated with the bootstrap boosted-paired Student's t-test.

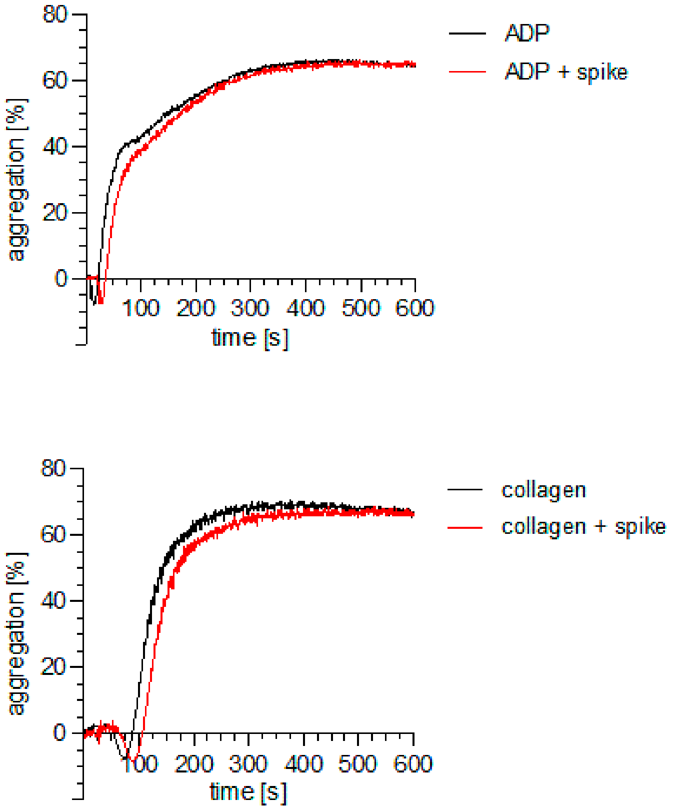

Figure S4. Representative curves of agonist-induced platelet aggregation measured in PRP by LTA. The effect of spike protein (2 µg/ml) on platelet aggregation was measured after PRP stimulation with 2 µmol/l ADP (top) or 1 µg/ml collagen (bottom).

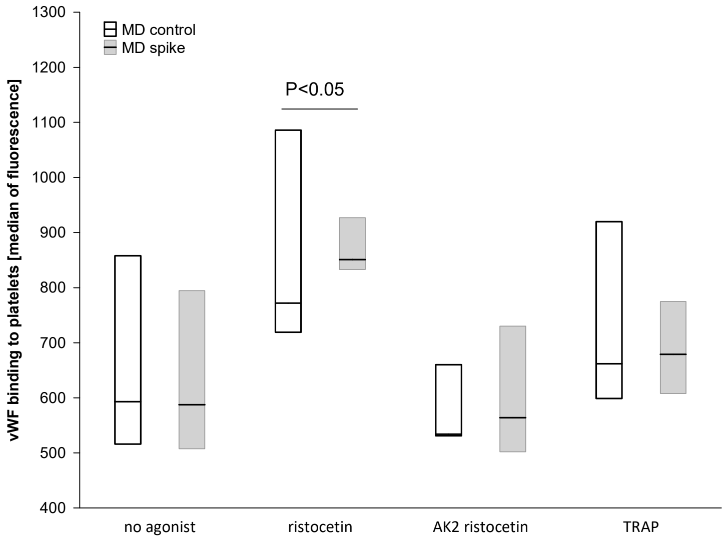

Figure S5. The binding of plasmatic vWF to the platelet surface in the presence of spike protein. Data are shown median and interquartile range (Q1; Q3). Spike protein significantly increased vWF binding to platelets compared to control in ristocetin-treated blood, P<0.05 (Wilcoxon signed-rank test, n=13).

Table S3. Effect of spike protein on thrombin generation in platelet poor plasma.

TG parameter	control	spike
Lag time [min]	8.1 ± 2.0	8.5 ± 2.3
Thrombin [nM]	344 ± 116	321 ± 127

Data shown as mean ± SD, n=43. Statistical significance of differences between control vs. spike sample was estimated with the paired Student's t-test. There was no significant difference between spike and control samples.

Table S4. Effect of spike protein on thrombin generation in platelet-rich plasma depending on tissue factor concentration.

	Thrombin [nM]		Lag [min]	
	control	spike	control	spike
TF 0	251 ± 94	205 ± 105	15.6 ± 3.7	14.9 ± 3.7
TF 1	236 ± 106	251 ± 97	15.8 ± 3.4	17.8 ± 3.5
TF 2.5	209 ± 111	232 ± 111	16.4 ± 4.4	16.0 ± 2.6
TF 5	235 ± 100	222 ± 94	15.4 ± 4.7	17.3.6

Data shown as mean ± SD, n=7. There were no significant differences between spike and control samples. TF was at the concentration of 0 – 1 – 2.5 – 5 pM. Statistical significance of differences between control vs. spike sample and between control vs. TF was estimated with Friedman test following post hoc analysis with Dunn's multiple comparison test. There was no significant difference between spike and control samples independent on TF presence.
